# Ectopic insulin secreting neuroendocrine tumor of kidney with recurrent hypoglycemia: a diagnostic dilemma

**DOI:** 10.1186/1472-6823-14-36

**Published:** 2014-04-17

**Authors:** S Ramkumar, Atul Dhingra, VP Jyotsna, Mohd Ashraf Ganie, Chandan J Das, Amlesh Seth, Mehar C Sharma, Chandra Sekhar Bal

**Affiliations:** 1 Department of Endocrinology and Metabolism, All India Institute of Medical Sciences, Ansari Nagar, New Delhi 110029, India; 2 Departments of Radiology, All India Institute of Medical Sciences, New Delhi, India; 3 Departments of Urology, All India Institute of Medical Sciences, New Delhi, India; 4 Departments of Pathology, All India Institute of Medical Sciences, New Delhi, India; 5 Departments of Nuclear Medicine, All India Institute of Medical Sciences, New Delhi, India

**Keywords:** Hyperinsulinemic hypoglycemia, Neuroendocrine tumour, Insulinoma, Carcinoid tumor, Renal tumour, 68Ga-Dotanoc scan, 99mTc-HYNICTOC scan

## Abstract

**Background:**

Hypoglycemia secondary to ectopic insulin secretion of non-pancreatic tumors is rare.

**Case presentation:**

We describe a middle aged woman with recurrent hypoglycemia. On evaluation, she was detected to have hyperinsulinemic hypoglycemia and right sided renal mass lesion. 68Ga-Dotanoc and 99mTc-HYNICTOC scans confirmed the intrarenal mass to be of neuroendocrine origin. Right nephrectomy was done and it turned out to be an insulin secreting neuroendocrine tumour. Neuroendocrine nature of this tumour was further confirmed by ultra-structural examination. Her hypoglycemia did not recur after resection of this tumour.

**Conclusion:**

Few cases of ectopic insulin secretion have been reported though some are not proven convincingly. This case addresses all the issues raised in previous case reports and proves by clinical, laboratory, functional imaging and immunohistochemical analysis that ectopic origin of insulin by non-pancreatic tumors does occur. To our knowledge, this is the first reported case of ectopic insulinoma arising from the kidney.

## Background

The most common cause of endogenous hypoglycemia is hyperinsulinemia secondary to islet cell tumours of pancreas. Hypoglycemia due to non-pancreatic tumours is infrequently reported. Most of these non-pancreatic tumours secrete factors with insulin like activity. Ectopic insulin secretion has been reported in few cases but not convincingly proved. We report a case of ectopic insulin secretion by neuroendocrine tumour (NET) of kidney. The ectopic origin of the tumour is demonstrated by functional imaging (^68^Ga-Dotanoc and ^99m^Tc-HYNICTOC), and by immunohistochemistry for insulin staining. We also extensively reviewed literature for similar cases of ectopic insulin secreting tumours published previously.

## Case presentation

This 44-year- old female was admitted for evaluation of recurrent hypoglycemic episodes of 3 years duration. She had complains of forgetfulness, altered behaviour, headache, palpitation, sweating, and giddiness. Most of her symptoms occurred early in the morning and these symptoms improved spontaneously in 15 minutes. She had consulted various physicians including neurologist and psychiatrist and had been diagnosed as pseudo-seizure, panic attacks or conversion disorder. One year back, she consulted endocrinologist and was diagnosed to have hypoglycemia and hyperinsulinemia. For suspecting insulinoma, she underwent CT abdomen which showed a right renal mass lesion and possibility of renal cell carcinoma was entertained. She was advised surgery for renal tumour. However, patient did not agree for surgery. There were no complains of hematuria, urinary frequency, urgency, burning micturition, diarrhoea or flushing episodes. She was not on antidiabetic treatment and was not hypertensive. For last 2 months, patient was having recurrent hypoglycemic seizures and came to this hospital.

After admission, she had persisted hypoglycemia and required continuous infusion of 10% Dextrose at the rate of 75 – 100 ml/hr in addition to 1-2 hourly feeds. Her hemogram, electrolytes, renal and liver functions were normal. HbA1c was 4.5%. Hormonal assay showed thyroxine (T4) 5.67 (5.1 – 14.1 mcg/dl), TSH 4.42 (0.27 – 4.2 mIU/ml), Insulin like Growth Factor- I(IGF-1) - 165.90 (62 – 205 ng/ml), growth hormone 1.20 ng/ml and cortisol 15.78 mcg/dl. Samples collected during hypoglycemia showed a serum insulin of 134 (2.6 – 24.9 μU/ml) and C-peptide of 13.35 (1.1 – 4.4) ng/ml. During another symptomatic hypoglycemia, she had plasma glucose of 44 mg/dl while her plasma insulin and C-peptide were 49.81 miu/ml and 9.36 ng/ml respectively confirming endogenous hyperinsulinemic hypoglycemia. She underwent high resolution radiological and functional imaging for localisation of insulinoma.

**Figure 1:**
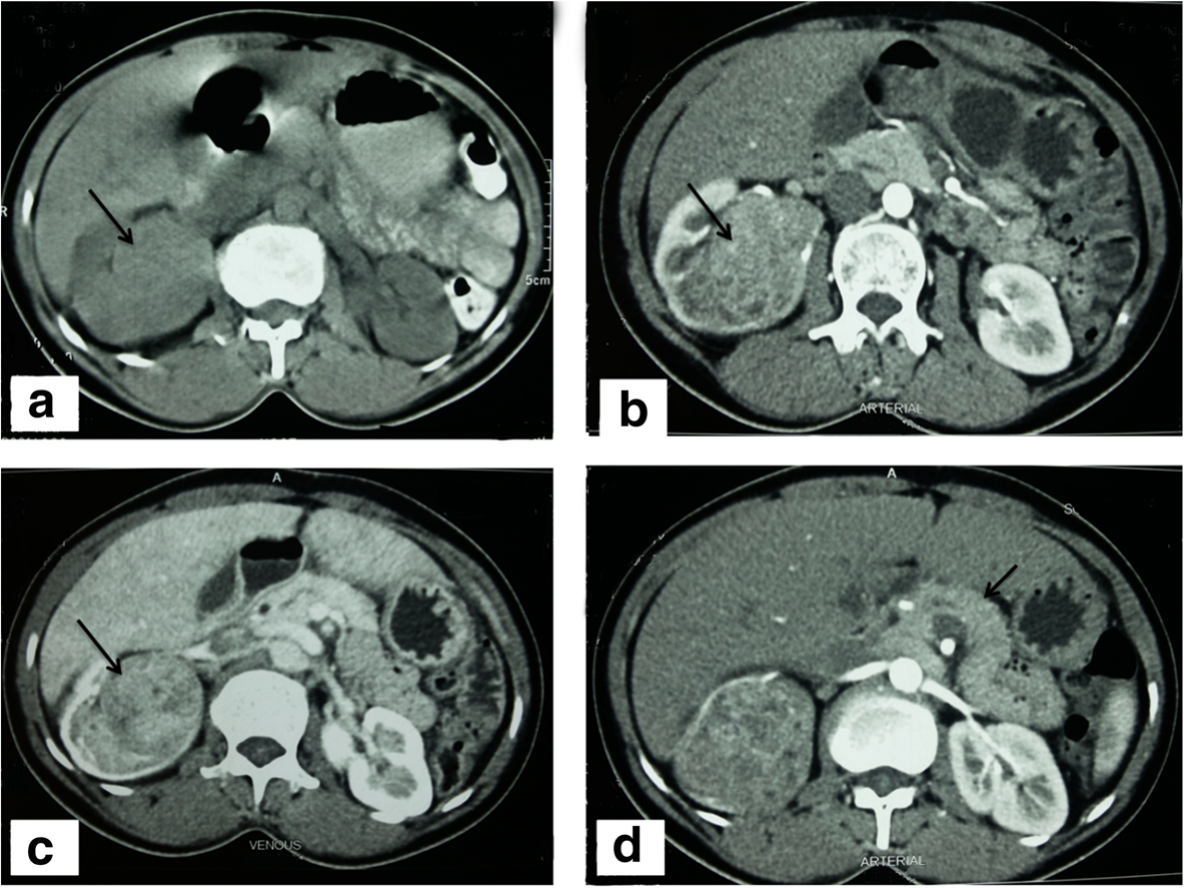
**CT scan.** Unenhanced CT axial image **(1a)** showing an isodense mass (arrow) to renal parenchyma which show enhancement in arterial phase image **(1b)** but lesser than the renal cortex. The enhancement continued till the venous phase **(1c)**. Arterial phase image **(1d)** taken at the level of pancreas (arrow) did not show any arterial enhancing lesion.

**Figure 2:**
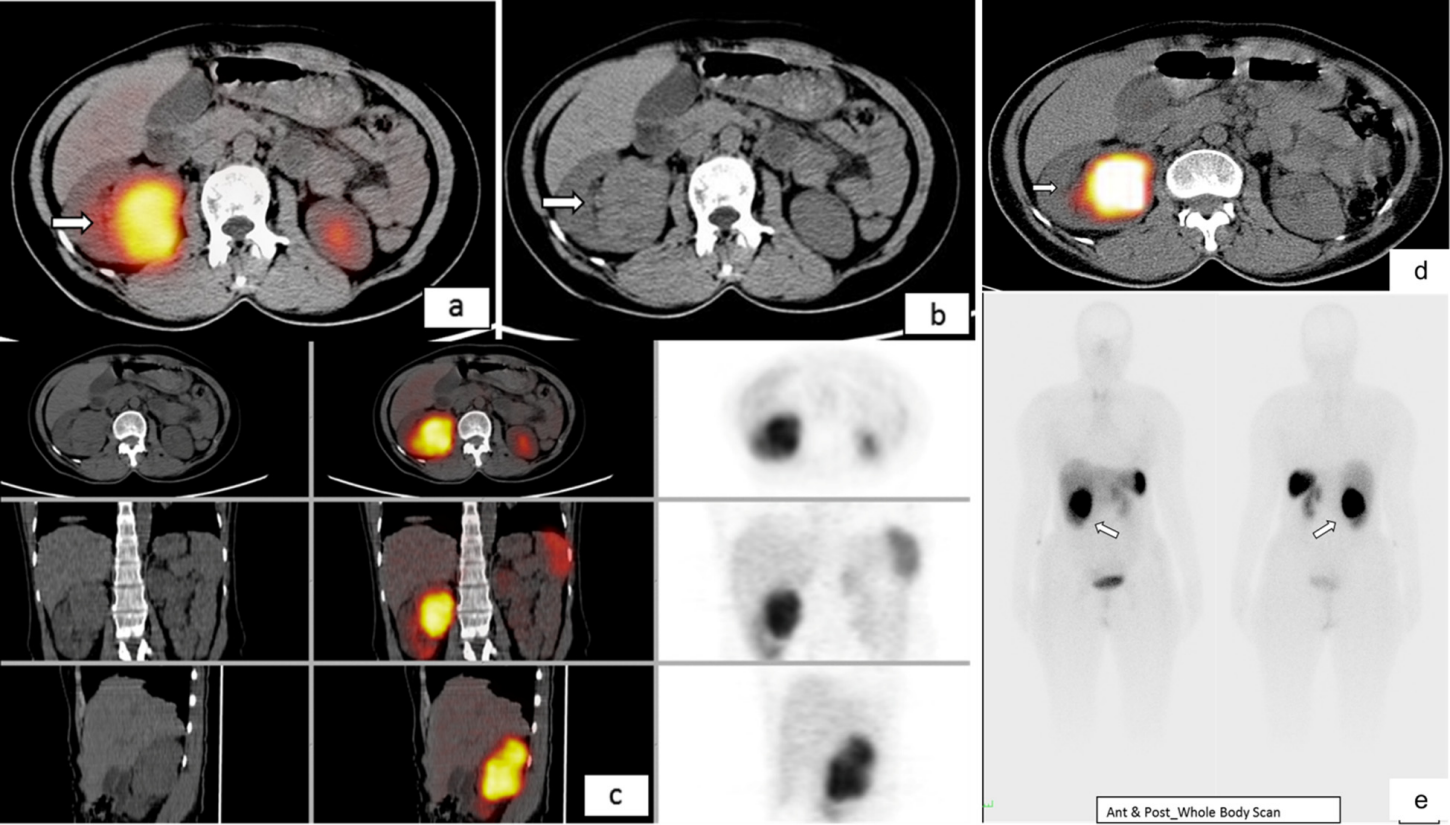
**Nuclear scans.** 3 mCi of 68Ga-Dotanoc PET/CT scan demonstrates somatostatin receptor (SSTR) expressing an intrarenal mass **(2a)** that corresponds to NCCT mass **(2b)**. There was no abnormal SSTR expressing tumour in pancreas **(2c)**, or any other abdominal structure. Similar observation was initially made from 15 mCi of 99mTc-HYNICTOC SPECT/CT scanning **(2d, 2e)**.

Both multi-phase CT abdomen (Figure 1) and contrast enhanced MRI abdomen demonstrated a mass lesion arising from upper- mid pole of right kidney (Figure 1a, 1b, 1c). No lesion was identified in the pancreas (Figure 1d). Endoscopic ultrasound also did not demonstrate any pancreatic lesion. 68Gallium DOTANOC scan (Figure 2a-c) and 99mTc-HYNICTOC (Figure 2d,e) did not demonstrate any uptake in the pancreas but both demonstrated intense uptake in the mass lesion indicating a NET. She underwent abdominal exploration and right nephrectomy. Post-operative period was uneventful and she became euglycemic without need for dextrose infusion. Her glucose values were maintained between 120 – 160 mg/dl thus confirming the right sided mass near renal hilum to be the source of ectopic insulin secretion.

### Pathologic examination

**Figure 3:**
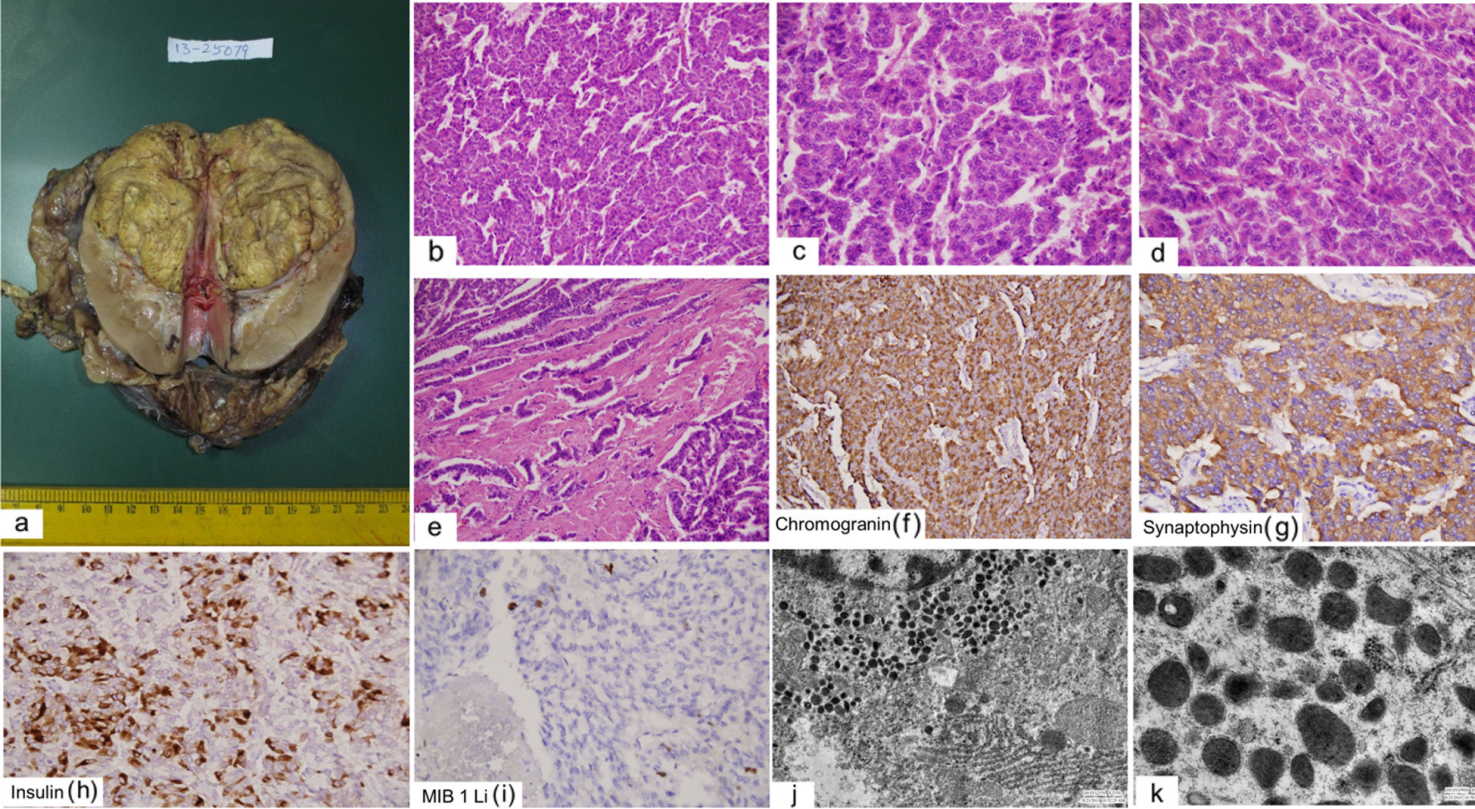
**Histopathology.** Gross photomicrograph showing a yellow colored well circumscribed tumour in the upper pole of the kidney **(3a)**. Photomicrographs showing diffuse and trabecular arrangement of tumour cells **(3b, 3c & 3d)** with marked desmoplastic reaction at places **(3e)**. (**3b**, **3c & 3d**: H&E × 400 each, **3e**: H&E × 200). Tumour cells are immunoreactive to chromogranin, synaptophysin and insulin (**3f**, **3g**, **3h** × 400 each). MIB 1 LI is 2% (d3i × 200). Electron micrographs showing numerous membrane bound electron dense neurosecretory granules, mitochondria and prominent rough endoplasmic reticulum (**3j** × 2550; **3k** × 9000 original magnification).

Gross examination: Grossly the kidney measured 10×7×6 cm and weighed 350 gram. A solid well circumscribed yellowish tumour, measuring 7×6×5 cm, was identified in the upper pole of kidney (Figure 3a). There was no capsule breach or renal sinus infiltration. Rest of the renal parenchyma and pelvicalyceal system were unremarkable. Areas of necrosis or hemorrhage were not identified.

Microscopic Finding: Microscopically, the tumor was composed of solid nests anastomosing cords and trabeculae of low columnar cells separated by a vascular stroma [Figures 3b-d]. The cellular outlines were indistinct with centrally placed oval nuclei, fine chromatin and inconspicuous nucleoli. The cytoplasm was eosinophilic, finely granular and moderate in amount. At places there were intense desmoplasia around the tumor cells (Figure 3e). Mitotic rate was 1 per 10 high power fields (hpf). Areas of tumour necrosis were not identified. Foci of perineural, lymphatic and vascular invasion were not seen. No intra or extra-cellular mucin was identified. Immunohistochemical staining revealed diffuse and intense staining for pancytokeratin, synaptophysin, chromogranin A, neuron specific enolase (NSE) and insulin (Figure 3f, 3g, 3h). MIB I labeling index was 2% (Figure 3i). The lymph nodes dissected from the specimen show metastasis (2/3).

Ultrastructural examination revealed numerous electron dense, membrane bound round to oval shaped granules of variable diameter in the cytoplasm [Figure 3j, 3k]. A narrow peripheral lighter zone was present in some of the granules.

Based on the above features; the diagnosis neuroendocrine tumour grade G1; pathological stage IV was entertained. Serum chromogranin A measured in the samples collected before surgery and stored in -40 degree was 2126.1 (N < 90.1 ng/ml).

### Follow up

No metastasis was detected. Our patient got discharged in stable condition on 10^th^ post-operative day. The patient is asymptomatic, euglycemic and disease free at last follow-up at 3 months.

## Discussion

Islet cell tumours of pancreas produce insulin which cause hypoglycemia. Hypoglycemia due to non-pancreatic tumours is infrequently reported and poorly understood. Various mechanisms were proposed [1] are: (a) insulin or insulin-like activity produced by the tumour, (b) decreased gluconeogenesis, (c) disruption of glucagon metabolism, and (d) increased utilization of glucose by the tumour. The non-suppressible Insulin like Activity (NSILA) has been purposed to be secondary to Insulin like Growth Factor-2. The non-islet cell tumours which commonly cause hypoglycaemia are of mesenchymal, epithelial or hematopoietic cell lines. Fibrosarcomas, mesotheliomas, leiomyosarcomas, and hemangiopericytomas are the most frequent types of tumours which cause hypoglycemia. Hepatoma, gastric, pancreatic, exocrine gland and lung carcinomas are epithelial cancers with frequent hypoglycemic potential. Mesenchymal and epithelial tumors, which generally present as large masses, are located in the mediastinum or the abdomen. These tumours are known to cause hypoglycemia via secretion of IGF-2 that leads to stimulation of insulin receptors [2]. In a series of 78 cases of non-islet-cell tumour, hypoglycemia (NICTH) due to IGF-2 production, hepatocellular carcinoma and gastric carcinoma were the common causes [3].

Ectopic insulin secreting tumours are rare, comprising only 1% to 2% of all insulinomas [4] and are commonly located in the peripancreatic or periduodenal region where most heterotopic pancreatic tissue is located. Ectopic insulin producing tumours located away from pancreatic beds are infrequently reported in literature. Only few cases were described in literature due to hyperinsulinemic hypoglycemia and non-pancreatic tumour and these tumors are described in Table 1.

**Table 1:** Insulin secreting extra-pancreatic tumors reported in literature

**Case**		**Evidence of hyperinsulinemia**^**a **^**in tumour**	**Resolution of hypoglycemia after resection of tumour**	**Evidence of neuro-endocrine origin**	**Mechanism of hypoglycemia -proposed**
1	Ovarian carcinoid [5]	Insulin staining (5%), EM – beta cell granules, absence of pancreatic tumor at autopsy	Not demonstrated	HPE	Direct tumoral secretion of insulin
2	Carcinoma cervix [6]	Insulin staining, absence of pancreatic tumor at autopsy	Not demonstrated	HPE	Liver metastasis^b^, Direct tumoral secretion of insulin
3	Bronchial carcinoid [7]	Insulin staining	Not demonstrated	HPE	Liver metastasis^c^, Direct tumoral secretion of insulin
4	Paraganglioma [8]	None	Yes	HPE	No conclusive evidence of direct tumoral secretion of insulin
5	Paraganglioma [9]	Insulin staining (3%)	Yes	HPE	Direct tumoral secretion of insulin
6	Pheochromocytoma [10]	Insulin stain negative, absence of pancreatic tumor at autopsy	Not demonstrated	HPE	Beta adrenoceptor mediated release of insulin from pancreas
7	Neuroendocrine tumor of liver [11]	Insulin staining, absence of any extrahepatic tumor at autopsy, Selective arterial calcium stimulation	Not demonstrated	HPE	Direct tumoral secretion of insulin^d^
8	Neuroendocrine tumour kidney (carcinoid) (present case)	Insulin staining, EM – beta cell granules,	Yes	HPE, 68Gallium DOTANOC, HYNICTOC imaging	Direct tumoral secretion of insulin

^a^Includes evidences other than biochemical documentation of hyperinsulinemia in critical samples(collected at the time of hypoglycemia).

^b^Liver functions are reportedly normal in this patient.

^c^70% of the liver was replaced by tumor at autopsy in this patient.

^d^This patient developed hypoglycemia after left hepatectomy.

HPE – Histo-pathological examination, EM – Electron Microscopy.

The patient under discussion had presented with right renal mass lesion and hyperinsulinemic hypoglycemia. We initially considered the possibility of incidentally detected right renal cell carcinoma in patient with insulinoma. In our Institute, we have both PET and SPECT imaging agents for octreoscan available for the localisation of insulinoma. Somatostain receptor scintigraphy (SRS) is an established functional imaging method for patients with NETs. The sensitivity of SRS for insulinoma is 50 – 60% [12]. We did both ^68^Ga-Dotanoc and ^99m^Tc-HYNICTOC scans for the localisation of insulinoma which unexpectedly revealed increased uptake in the right renal mass lesion itself confirming it to be a NET. To the best of our knowledge, this is the first case of ectopic insulin secretion confirmed *in vivo* by ^68^Ga-Dotanoc and ^99m^Tc-HYNICTOC scan. Further, the neuroendocrine and insulin secreting nature of the tumour was confirmed *ex vivo* by histopathological examination, immunohistochemistry and ultra-structural examination. Paraganglioma and pheochromoctyoma were also reported to cause hyperinsulinemic hypoglycemia and also show tracer uptake by SRS and are immunoreactive for chromogranin A and syanptophysin. Though the mass lesion was arising from upper pole of right kidney, it was intra-renal and hisopathological examination was suggestive of NET.

NETs are neoplasms that arise from cells of dispersed neuroendocrine system. Although there are many kinds of NETs but they are treated as a group of tumours as these neoplasms share common features such as histology, immunoreactivity for neuroendocrine markers (chromogranin A, synaptophysin, neuron specific enolase), presence of neurosecretory granules, and secretion of biogenic amines and polypeptide hormones. Neuroendocrine tumours arising from pancreas are classified by the hormone most commonly secreted. Neuroendocrine tumours arising from the intestine, respiratory system and rest of the body were known as carcinoids but under the recent nomenclature they are all know as neuroendocrine neoplasm (NEN) of tumour. Primary carcinoid tumours of kidney are extremely rare and around 90 cases has been reported in literature [13]. None of the reported cases including two cases were reported from our institute previously [14], [15] were insulin secreting.

The cell of origin of renal carcinoid is unclear as neuroendocrine cells are not normally found in adult renal parenchyma. Carcinoid tumours of kidney are usually asymptomatic. In symptomatic cases, these tumours present with abdominal pain or abdominal mass with hematuria or fever. Evidence of carcinoid syndrome with serotonin-related flushing, generalized edema and diarrhoea, and occasional elevation of urine 5-hydroxyindoleaceticacid are uncommon and are seen in less than 10% of cases [14]. Our patient did not have these symptoms prior to presentation, although she had flushing, edema and blanching of the skin intraoperatively. Rarely, these tumours may present with neuroendocrine syndromes like cushing syndrome, vipoma, or glucagonoma [16]. Our case presented as insulinoma. Macroscopically, renal carcinoid tumours are usually solitary and unilateral. They are well circumscribed with a lobulated and bulging appearance. The cut surface the tumour is yellow-tan, or red brown as also observed in the case under discussion. Renal carcinoids exhibit histological features that are typical of carcinoids at other sites. Primary carcinoid tumors as well as metastasis possess high affinity receptors for somatostatin in 87% of cases [17]. Localization of gastrointestinal tract carcinoid tumours and pancreatic endocrine tumors has been achieved by the use of radiolabeled octreotide, a synthetic and slowly degraded somatostatin analogue that has a high affinity for somatostatin receptors. Use of indium-111 pentetreotide scanning in the diagnosis of carcinoid tumors had been reported [18]. In the present case, functional imaging with ^68^Ga-Dotanoc and ^99m^Tc-HYNICTOC was done for the localisation of insulinoma. Both showed uptake in the right renal mass lesion and no uptake in pancreas (Figure 2a-e). All available modalities for diagnosis of carcinoid tumors were employed namely Electron microscopy, immunohistochemistry, both PET and SPECT octreotide scan along with conventional radiographic imaging techniques were used in preoperative diagnosis this tumour.

Prognosis of NET depends upon the grade and stage of the tumour. Although the grade of the tumour was low, however stage was high as there was metastasis in the regional lymph nodes. There was no recurrence of hypoglycaemia on till this time of reporting, however, long-term close follow up is needed for the malignant behaviour.

## Conclusions

Ectopic insulin secreting extra-pancreatic tumours are rare. Confirmation of the source of hyperinsulinemia is often difficult. To our knowledge, this is the first case of extra-pancreatic insulin secreting neuroendocrine tumour fully characterised by biochemical, radiological & functional imaging, histopathology and immunohistochemistry. Our patient had successful post-operative outcome and maintained euglycaemia after three months of follow-up.

## Consent

Written informed consent was obtained from the patient for publication of this Case report and any accompanying images. A copy of the written consent is available for review by the Editor of this journal.

## Abbreviations

SRS: Somatostain receptor scintigraphy; NEN: Neuroendocrine neoplasm; NICTH: Non-islet-cell tumour hypoglycemia; NET: Neuroendocrine tumor.

## Competing interests

The authors declare that they have no competing interests.

## Authors’ contributions

1) RS, JVP, DA, GMA have managed the case clinically with the help of DCJ (radiology), BCS (Nuclear Medicine), SMC (pathology) and SA (Uro-Surgery) for diagnosis and management. DCJ has done and reported the CT scans, BCS has done and reported the Nuclear scans (68Ga-Dotanoc PET/CT and 99mTc-HYNICTOC SPECT/CT scans), SMC has done the ultrastructural examination and immunostaining while SA has performed the definitive surgery which was curative in this case. 2) RS And DA have been involved in drafting the manuscript and JVP in revising it critically for important intellectual content. 3) All authors have given final approval of the version to be published.

## Pre-publication history

The pre-publication history for this paper can be accessed here:

http://www.biomedcentral.com/1472-6823/14/36/prepub
